# Full-Range Public Health Leadership, Part 1: Quantitative Analysis

**DOI:** 10.3389/fpubh.2015.00073

**Published:** 2015-04-30

**Authors:** Erik L. Carlton, James W. Holsinger, Martha Riddell, Heather Bush

**Affiliations:** ^1^Division of Health Systems Management and Policy, University of Memphis, Memphis, TN, USA; ^2^Department of Preventive Medicine, University of Kentucky, Lexington, KY, USA; ^3^Department of Health Management and Policy, University of Kentucky, Lexington, KY, USA; ^4^Department of Biostatistics, University of Kentucky, Lexington, KY, USA

**Keywords:** public health leadership, multifactor leadership questionnaire, public health workforce development, transformational leadership, local health department, full-range leadership

## Abstract

**Background:**

Workforce and leadership development are central to the future of public health. However, public health has been slow to translate and apply leadership models from other professions and to incorporate local perspectives in understanding public health leadership.

**Purpose:**

This study utilized the full-range leadership model in order to examine public health leadership. Specifically, it sought to measure leadership styles among local health department directors and to understand the context of leadership in local health departments.

**Methods:**

Leadership styles among local health department directors (*n* = 13) were examined using survey methodology. Quantitative analysis methods included descriptive statistics, boxplots, and Pearson bivariate correlations using SPSS v18.0.

**Findings:**

Self-reported leadership styles were highly correlated to leadership outcomes at the organizational level. However, they were not related to county health rankings. Results suggest the preeminence of leader behaviors and providing individual consideration to staff as compared to idealized attributes of leaders, intellectual stimulation, or inspirational motivation.

**Implications:**

Holistic leadership assessment instruments such as the multifactor leadership questionnaire can be useful in assessing public health leaders’ approaches and outcomes. Comprehensive, 360-degree reviews may be especially helpful. Further research is needed to examine the effectiveness of public health leadership development models, as well as the extent that public health leadership impacts public health outcomes.

## Full-Range Public Health Leadership

Today’s public health leaders face increasingly complex challenges while being called on more and more to collaborate with and lead in the communities in which they live, work, and serve. Health care reform is transforming the entire health system, including public health. Public health agencies, tasked with basic care for the indigent and assurance of health standards for whole populations, have their budget appropriations cut so that they not only have reduced physical and medical resources, but also have fewer human resources to assign to meet those needs (1, 2). Those human resources – the public health workforce – are in the midst of significant upheaval. The retirement of large numbers of highly experienced members of the public health workforce results in a dearth of practical knowledge as new staff members, when they can be hired, may not have similar educational or experiential backgrounds (3). Concern for what national health reform will mean for the public health organization and its employees is an issue (4, 5). Committed and effective leadership in public health has perhaps never been more important (6). The vital issues of today demand public health leaders who are as skilled and astute politically as they are at managing the technical and systemic aspects of public health (7, 8). Given this highly dynamic context, understanding and developing the leadership abilities of public health leaders is essential to meeting the demands of population health issues.

In *The Future of Public Health* (9), the Institute of Medicine (IOM) suggested that without appropriate attention to workforce and leadership development, public health organizations would be ill prepared to fulfill the essential purposes of public health. By 2003, the IOM had published two additional reports, *The Future of the Public’s Health in the 21st Century* (10) and *Who Will Keep the Public Healthy?* (11). Both of these studies reiterated the workforce and leadership development themes identified 15 years previously: leadership training and development activities must be a priority for both governmental public health organizations and academic public health institutions.

### Public health leadership development efforts

Since the initial IOM report, public health practice and academic organizations have focused increasingly on public health leadership development. These efforts include the development of state, regional, national, and international public health leadership institutes, the formation of a national public health leadership development network, and the development of public health leadership competency frameworks for both educational and practice settings. These efforts shape the development of public health leaders now and into the future.

To assist in the shaping of future public health leaders, the Association of Schools and Programs of Public Health (ASPPH) has developed core competencies for individuals obtaining Master of Public Health (MPH) (12) or Doctor of Public Health (DrPH) (13) degrees. Both models delineate specific competencies necessary for public health students to evince in order to achieve their leadership potential. Further, both models define leadership in terms of creating a shared vision, combined with notions of motivating others, galvanizing organizational and community resources to address public health problems, and utilizing the best strategies and practices to enhance service and solve problems. Finally, both models highlight the potential of public health graduates to lead organizations and communities, with the competence to influence others, establish a shared vision, and accomplish the mission of public health. Further, leadership competencies are not the purview of ASPPH alone. The Core Competencies for Public Health Professionals (14) include leadership and systems thinking skills. Clearly, leadership competence is on the agenda for current and future public health workforce efforts.

The overarching theme of public health leadership literature and public health leadership development efforts is the need for highly skilled and well-educated leaders capable of galvanizing organizations and communities in transformational change processes that not only ensure, but also seek to improve the health and well-being of the public. These efforts frame a vision for public health leadership that currently prefers transformational, change-agent leaders. However, as Nicola (15) has pointed out, classic management functions – planning, organizing, leading, and controlling – remain vital to assuring the performance of public health organizations. While transformational leadership qualities enable public health leaders to engage communities in efforts to improve population health, the full range of leadership qualities, including technical and managerial acumen, is necessary not only to lead change but also to effectively attend to general and regular organizational tasks and responsibilities should not be overlooked.

To that end, leadership may have discipline-specific requirements unique to public health. Rather than assume that leadership qualities, characteristics, and processes are universal to all professions, public health agencies would benefit from a better understanding of the skills and competencies required for successful public health leadership.

### Purpose

The purpose of this study was to examine the full range of leadership styles among local health department directors in Kentucky. Specifically, this portion of the study quantitatively explores: (1) the leadership styles of local health department directors and their perceptions of organizational outcomes, (2) the sub-components of each style contributing to the overall leadership style of local health department directors, and (3) whether there is any relationship between leadership styles and specific county health outcomes. We posited that while leadership styles would vary, the predominant leadership style, and the one most closely correlated with positive leadership outcomes, would be the transformational leadership style.

## Method

This study used the multifactoral leadership questionnaire (MLQ), developed by Avolio and Bass (16). The 45-item MLQ is among the most commonly used and validated measures of full-range leadership styles (16–23), and has been shown to be an effective tool in leadership development (24, 25). The MLQ measures three general leadership styles – transformational, transactional, and passive-avoidant – and nine sub-types (see Table 1), as well as outcomes of leadership. Each of the individual leadership components in the MLQ, including the leadership outcomes, yields a raw score between 0 and 4. These scores are translated into percentiles based on national norms for self-reported data provided with the MLQ instrument. Licenses to use the MLQ were purchased. The MLQ was combined with demographic variables and distributed electronically to local public health directors using Qualtrics.

**Table 1:** **Brief definitions of leadership types and sub-types and outcomes of leadership**.

Leadership type	Definition/characteristics
Transformational leadership	Transformational leaders influence and change followers’ awareness of what is important, providing a greater vision of themselves and the opportunities and challenges of their environment. They are proactive and strive to optimize individual, group, and organizational development, and innovation. They influence associates, coworkers, and followers to strive for higher levels of performance and higher moral and ethical standards
Idealized influence attributes (IIA)	Idealized attributes include: instilling pride in others, going beyond self-interest for the good of the group, acting in ways that build others’ respect, and displaying a sense of power and confidence
Idealized influence behaviors (IIB)	Idealized behaviors include: talking about important values and beliefs, specifying the importance of having a strong sense of purpose, considering the moral and ethical consequences of decisions, and emphasizing the importance of having a collective sense of mission
Inspirational motivation (IM)	These leaders behave in ways that motivate others by providing meaning and challenge to their followers’ work. Enthusiasm and optimism arouse individual/team spirit. They articulate a compelling vision of the future and expressing confidence that goals will be achieved
Intellectual stimulation (IS)	These leaders stimulate their followers’ efforts to be innovative and creative by questioning assumptions, reframing problems, and approaching old situations in new ways. They re-examine critical assumptions, seek differing perspectives when solving problems, get others to look at problems from many different angles, and suggest new ways of looking at how to complete assignments
Individual consideration (IC)	These leaders pay attention to each individual’s need for achievement and growth. Individual differences in terms of needs and desires are recognized. These leaders spend time teaching and coaching and help others to develop their strengths
Transactional leadership	Transactional leaders focus on constructive (contingent reward) and corrective (management-by-exception) transactions, by defining expectations and promoting performance to achieve these levels. These leadership styles are among the core “management” functions in organizations
Contingent reward (CR)	These leaders clarify expectations and offer recognition when goals are achieved. These leaders provide others with assistance in exchange for their efforts, discuss in specific terms responsibility for achieving performance targets, make clear what one can expect to receive when performance goals are achieved
Management-by-exception: active (MBEA)	This style of leadership implies closely monitoring for deviances, mistakes, and errors and then taking corrective action as quickly as possible when they occur. These leaders focus attention on irregularities, mistakes, exceptions, and deviations from standards
Passive-avoidant leadership	Passive leaders do not specify agreements, clarify expectations, or provide goals and standards to be achieved by followers. It is a style typified as being more passive and reactive
Management-by-exception: passive (MBEP)	Passive leaders fail to interfere until problems become serious, waiting for things to go wrong before taking action. They show a firm belief in “if it ain’t broke, don’t fix it”
Laissez-Faire (LF)	Laissez-faire leaders avoid getting involved when important issues arise, are often absent when needed, avoid making decisions, and delay responding to urgent questions

Using a consensus-driven sampling approach, this study identified local health directors as potential subjects by interviewing key state and university public health leaders. Specifically, one paragraph, literature-based (i.e., theory-driven) descriptions of transformational and transactional leadership styles were given to an expert group of individuals well-acquainted with potential study participants. These individuals were independently asked to identify approximately five to seven effective local health department directors who possessed qualities of either transformational leadership or transactional leadership. Passive-avoidant leadership is also referred to as “non-leadership” in the full-range leadership literature. For this reason, we sought to sample only leaders thought to possess one of the two primary leadership styles – transformational and transactional. A group of 15 transformational directors and 15 transactional directors were identified from which a sample of 10 directors from each leadership style category was randomly selected. This random selection served to reduce sampling bias. Some of the health department directors declined participation in the study. While completion of the survey was voluntary, initial non-response initiated two additional attempts to solicit participation in order to maximize the response rate. All surveys were administered electronically using Qualtrics, with links to the survey provided by email to the participants. Finally, as an incentive to participate, individual directors who elected to complete the survey received direct feedback in the form of a report that interpreted the results of survey, identifying leadership strengths, and suggesting potential growth areas.

A final sample of 20 directors was identified and they were invited to participate in the quantitative phase of the study. The sample consisted of 10 directors perceived to be more transformational and 10 directors perceived to be more transactional. To encourage participation, the survey email was preceded by an email from the state commissioner for public health. The survey email was followed by up to two additional contacts inviting participants to complete the survey. Thirteen directors completed the survey for a 65% response rate. This included seven directors from the perceived transformational group and six from the perceived transactional group.

### Leadership outcomes

Transformational and transactional leadership are both related to the success of the group. Success, or outcomes of leadership, was measured with the MLQ through leaders’ self-reported skills at motivation, effectiveness in interacting at different levels of the organization, and perceived employee satisfaction with leaders’ methods of working with others. These include: extra effort (EE), effectiveness (EFF), and satisfaction with the leadership (SAT). EE may be defined as the extent to which leaders get others to do more than they expected to do, heighten others’ desire to succeed, and increase others’ willingness to try harder. Effective leaders are effective in meeting others’ job-related needs, in representing their group to higher authority, and in meeting organizational requirements. They lead groups that are effective. Leadership satisfaction includes: using methods of leadership that are satisfying and working with others in a satisfactory way.

### Analysis

The extent of transformational and transactional leadership styles and components of leadership styles reported by study participants was explored with descriptive statistics (e.g., means and SDs). Pearson bivariate correlation coefficients were calculated to measure relationships between variables of interest. Given the limited sample size, power analyses, as well as additional or more complex analyses, were not feasible. Finally, findings from the descriptive analysis were examined for any relationship to existing county-level data: Beale Codes, which measure relative population density on a rural-urban continuum, and the County Health Rankings (26), which rank counties based on health outcomes (mortality and morbidity) and health factors (health behaviors, clinical care, physical environment, and social and economic factors).

This study was approved by the University of Kentucky Institutional Review Board.

## Results

### Participant demographics

Thirteen local health directors completed the initial survey phase of the study. Complete demographics are provided in Table 2 below. Nearly two-thirds (62%) of participants were female and the majority (*n* = 12, 92%) were white. Participants were of varying ages. All participants had at least a bachelor’s degree. The majority (*n* = 11, 85%) classified their health departments as rural and the others (*n* = 2, 15%) classified their health departments as sub-urban.

**Table 2:** **Demographics (*n* = 13)**.

Background information	Participants (*n* = 13)
Gender
Male	5 (38%)
Female	8 (62%)
Race
White/Caucasian	12 (92%)
Black	1 (8%)
Other	0 (0%)
Age
18–25	0 (0%)
26–35	1 (8%)
36–45	2 (15%)
46–55	7 (54%)
55 +	3 (23%)
Highest education completed
High school/Associate’s degree	0 (0%)
Bachelor’s degree	2 (15%)
Master’s degree	10 (77%)
Doctoral degree	1 (8%)
Public health degree (MPH, DrPH)
Yes	4 (31%)
No	9 (69%)
Graduate of a ph leadership institute
Yes	9 (69%)
No	4 (31%)
Type of health department
Urban	0 (0%)
Sub-urban	2 (15%)
Rural	11 (85%)

Personal leadership development was at least a moderate priority for participants. About a third of participants (*n* = 4, 31%) indicated that their own leadership development was a moderate priority. Just over two-thirds (*n* = 9, 69%) felt it was a high priority. Similarly, developing the leadership abilities of staff was also at least a moderate priority for participants. The results were identical. About a third of participants (*n* = 4, 31%) indicated that their own leadership development was a moderate priority. Just over two-thirds (*n* = 9, 69%) felt it was a high priority. Finally, participants felt their leadership at least had considerable influence on the performance of the organization. Just over two-thirds of participants (*n* = 9, 69%) believed their leadership had considerable influence on the performance of the organization; while about a third (*n* = 4, 31%) indicated that their own leadership had significant influence on organizational performance.

### Self-reported leadership styles

Table 3 provides a reference to the reader on the major leadership styles and their sub-style components.

**Table 3:** **Leadership styles and sub-styles**.

Transformational		Transactional		Passive-avoidant	
1.	Idealized attributes	1.	Contingent reward	1.	Management by exception – passive
2.	Idealized behaviors	2.	Management by exception – active	
3.	Inspirational motivation			2.	Laissez-Faire
4.	Intellectual stimulation		
5.	Individual consideration		

Participants self-reported a wide range of leadership styles, with wide variance across all leadership characteristics.

#### Transformational Leadership

Among characteristics of transformational leadership, idealized attributes had a mean percentile of 53.15 (SD = 33.63), with a minimum percentile of 1 and a maximum percentile of 90. Idealized behaviors had a mean percentile of 74.23 (SD = 20.50), with a minimum percentile of 30 and a maximum of 95. Inspirational motivation had a mean percentile of 55.00 (SD = 29.51), with a minimum percentile of 20 and a maximum of 95. Intellectual stimulation had a mean percentile of 66.92 (SD = 19.21), with a minimum percentile of 40 and a maximum of 95. Finally, individual consideration had a mean percentile of 64.23 (SD = 27.37), with a minimum percentile of 10 and a maximum of 95.

#### Transactional Leadership

Among characteristics of transactional leadership, contingent reward had a mean percentile of 61.92 (SD = 25.21), with a minimum percentile of 30 and a maximum percentile of 95. Management by exception – active had a mean percentile of 38.15 (SD = 31.88), with a minimum percentile of 5 and a maximum of 80.

#### Passive-Avoidant Leadership

Among characteristics of passive-avoidant leadership, management by exception – passive had a mean percentile of 38.15 (SD = 26.93), with a minimum percentile of 1 and a maximum percentile of 95. Laissez-Faire had a mean percentile of 30.54 (SD = 36.44), with a minimum percentile of 1 and a maximum of 95.

### Self-reported leadership outcomes

Consistent with the range of self-reported leadership styles, participants also reported variance in leadership outcomes, especially for EFF. EE had a mean percentile of 62.7 (SD = 18.34), with a minimum percentile of 26 and a maximum percentile of 88. EFF had a mean percentile of 48.9 (SD = 20.73), with a minimum percentile of 20 and a maximum of 88. Finally, satisfaction had a mean percentile of 34.4 (SD = 29.30), with a minimum percentile of 1 and a maximum of 95. Finally, overall work outcome had a mean percentile of 34.4 (SD = 29.30), with a minimum percentile of 1 and a maximum of 95.

### Bivariate correlations for self-reported leadership styles and outcomes of leadership

#### Transformational Leadership

When individual components of transformational leadership were examined, strong and significant correlations were found among most components and overall transformational (OTF) leadership, except for intellectual stimulation. As shown in Table 4, intellectual stimulation was not significantly correlated to any of the other individual components of transformational leadership or OTF leadership. Further, idealized attributes were only strongly and significantly correlated with idealized behaviors (Pearson *r* = 0.621, *p* < 0.05) and OTF leadership (Pearson *r* = 0.641, *p* < 0.05), and not the other components. Idealized behaviors were strongly and significantly correlated with inspirational motivation (Pearson *r* = 0.782, *p* < 0.01) and individual consideration (Pearson *r* = 0.652, *p* < 0.05), as well as OTF leadership (Pearson *r* = 0.914, *p* < 0.001). Among attributes of transformational leadership, idealized behaviors (Pearson *r* = 0.914, *p* < 0.001) and inspirational motivation (Pearson *r* = 0.834, *p* < 0.001) were most strongly and most significantly correlated with OTF leadership.

**Table 4:** **Pearson correlation coefficients for components of transformational leadership (*n* = 13)**.

	IA	IB	IM	IS	IC	OTF
Idealized attributes (IA)	–	0.621*	0.472	−0.249	0.120	0.641*
Idealized behaviors (IB)		–	0.782**	0.078	0.652*	0.914***
Inspirational motivation (IM)			–	0.129	0.459	0.834***
Intellectual stimulation (IS)				–	0.518	0.332
Individual consideration (IC)					–	0.745**
Overall transformational (OTF)						–

*p < 0.05. **p < 0.01. ***p < 0.001.

#### Transactional Leadership

As shown in Table 5, among attributes of transactional leadership, both contingent reward (Pearson *r* = 0.640, *p* < 0.05) and management by exception – active (Pearson *r* = 0.794, *p* < 0.01) were strongly and significantly correlated with overall transactional (OTA) leadership. However, no correlation was found between the individual components.

**Table 5:** **Pearson correlation coefficients for components of transactional leadership (*n* = 13)**.

	CR	MBEA	OTA
Contingent reward (CR)	–	0.042	0.640*
Management by exception – active (MBEA)		–	0.794**
Overall transactional (OTA)			–

*p < 0.05. **p < 0.01.

#### Passive-Avoidant Leadership

Strong and significant correlations were found among components of passive-avoidant leadership. Specifically, both management by exception – passive (Pearson *r* = 0.897, *p* < 0.001) and Laissez-Faire (Pearson *r* = 0.945, *p* < 0.001) were strongly and significantly correlated with overall passive-avoidant (OPA) leadership. Additionally, a strong and significant correlation (Pearson *r* = 0.704, *p* < 0.01) was also found between the two individual sub-scale components (see Table 6).

**Table 6:** **Pearson correlation coefficients for components of passive-avoidant leadership (*n* = 13)**.

	MBEP	LF	OPA
Management by exception – passive (MBEP)	–	0.704**	0.897***
Laissez-Faire (LF)		–	0.945***
Overall passive-avoidant (OPA)			–

**p < 0.01. ***p < 0.001.

#### Overall Leadership Styles and Outcomes of Leadership

As shown in Table 7, no correlations existed between overall leadership styles. However, strong and significant correlations were found among OTF leadership styles and outcomes of leadership, including overall outcomes of leadership. Specifically, OTF leadership was strongly and significantly correlated to EE (Pearson *r* = 0.818, *p* < 0.01), EFF (Pearson *r* = 0.759, *p* < 0.01), and satisfaction (Pearson *r* = 0.845, *p* < 0.001), as well as overall leadership outcomes (Pearson *r* = 0.846, *p* < 0.001). Further, as shown below, strong and significant correlations were also found among all individual components of leadership outcomes, as well as overall leadership outcomes.

**Table 7:** **Pearson correlation coefficients for overall leadership styles and leadership outcomes (*n* = 13)**.

	OTF	OTA	OPA	EE	EFF	SAT	LO
Overall transformational (OTF)	–	−0.010	−0.328	0.818**	0.759**	0.845***	0.846***
Overall transactional (OTA)		–	−0.003	−0.003	0.101	0.252	0.123
Overall passive-avoidant (OPA)			–	−0.265	−0.166	−0.341	−0.265
Extra effort (EE)				–	0.839***	0.867***	0.944***
Effectiveness (EFF)					–	0.859***	0.954***
Satisfaction (SAT)						–	0.953***
L’ship outcomes (LO)							–

**p < 0.01. ***p < 0.001.

### Bivariate correlations for self-reported leadership styles and county health outcomes

As shown in Table 8, neither participants’ leadership styles nor individual leadership style components were correlated with County Health Rankings (health outcomes and health factors). However, significant moderate correlations were found between county Beale code classification and both health outcomes (Pearson *r* = 0.401, *p* < 0.01) and health factors (Pearson *r* = 0.313, *p* < 0.05). Further, health outcomes were strongly and significantly correlated with health factors (Pearson *r* = 0.683, *p* < 0.01). Finally, when calculated across the counties they serve, a strong, significant, and negative correlation (Pearson *r* = .-0.608, *p* < 0.001) was found between participants’ OTF leadership and overall passive-avoidant leadership styles.

**Table 8:** **Pearson correlation coefficients for leadership styles, county location, and county health rankings (*n* = 45)**.

	OTF	OTA	OPA	BC	HO	HF
Overall transformational (OTF)	–	−0.099	−0.608***	−0.195	−0.160	0.150
Overall transactional (OTA)		–	−0.019	−0.174	0.112	−0.022
Overall passive-avoidant (OPA)			–	0.287	0.220	−0.086
Beale code (BC)				–	0.401**	0.313*
Health outcomes (HO)					–	0.683**
Health factors (HF)						–

*p < 0.05. **p < 0.01. ***p < 0.001.

## Discussion, Implications, and Limitations

The results indicate that among the sample studied, leadership outcomes were a function of transformational rather than transactional leadership. The high degree of significant correlation between transformational leadership and self-reported outcome constructs such as EE, EFF, and satisfaction suggests a perception among public health leaders that such transformational qualities can lead to desirable performance outcomes, including better operated health departments, and harder working, more satisfied employees.

As a consequence, an examination of the individual transformational leadership sub-scales is compelling. Among the leaders sampled, transformational leadership was not a function of intellectual stimulation and was only mildly, if significantly, correlated with idealized leader attributes. Rather, the idealized behavior, inspirational motivation, and individual consideration components of the transformational leader stand out. It is possible that those surveyed viewed these aspects of leadership more positively, or perceived that they are leadership skills in which these leaders feel strong or confident and so are qualities more fully relied upon. Certainly, most leaders will not possess all sub-components of a given leadership style; however, a defined group is leaders commonly identified a discrete set of leadership qualities is intriguing and likely merits further examination.

Leadership outcomes that were not correlated with passive-avoidant leadership (also known as non-leadership) were expected and it is not surprising that leadership styles were not associated with country health rankings. Perhaps, if all local health department executives in a state were surveyed and their leadership style(s) compared to the relative health ranking of the county(ies) for which they are responsible, some correlation maybe found; however, that is not likely. These rankings are broad measures of health within communities and are largely a function of factors much broader than the individual at the helm of a local health department, whose tenure in that position is likely not nearly as long as the time required to either directly cause or remedy the noted rankings and the health outcomes on which they are based.

### Implications for practice

While fuller implications of both quantitative and qualitative phases of this study will be discussed in the adjoining companion paper, this quantitative portion of the study has important implications for public health practitioners. The first of these is the potential for public health leaders, including local health department directors and local boards of health, as well as public health educators, to adopt well-grounded and more holistic instrumentation such as the MLQ in their assessment of leadership styles and abilities. Such tools provide and, if used generatively, help develop greater self-awareness related to leadership styles and behaviors. Many leadership style and personality instruments have been developed, including but certainly not limited to the MLQ instrument used in this study. These instruments are available to measure a range of skills from basic management to levels of emotional intelligence. Developing a detailed leadership assessment for public health practitioners may be useful if based on empirically validated instruments, including a self-generating interpretive report which could be made readily available to interested health departments or individuals. These tools may be especially useful when orienting new supervisors or as a part of accredited higher education programs.

Assessments such as the MLQ may be helpful to state health officials and for local boards of health that may be involved in the selection and development of new local public health directors. Many members of local health boards are not trained in public health and may be appointed to fill codified positions on the local board; yet, they are tasked with hiring and supervising local directors, which may have a significant positive or negative impact on the public health agency. Boards of health should not only account for the health needs of their communities, but should also consider carefully the qualities and aspects of leaders they desire in their health director. While basic comprehension of public health services and systems may be fundamental as a qualification, if board members want a director to merely manage the books and tend to the basic tasks of clinical and environment public health, or if the health department may not have a fiscal or political environment conducive to innovation and change, they should look for a more transactional leader. If, however, the board is interested in a director who will challenge the *status quo* by finding creative and innovative ways to improve population health, they should focus their candidate search on more transformational leaders. Providing board members with simple measurement tools to identify these leadership styles should be helpful in this process.

### Implications for research

A larger, more comprehensive, and randomized study utilizing 360-degree reviews by supervisors, colleagues, and subordinates could improve the measurement of public health leaders and the understanding of the qualities and characteristics that may contribute to effective public health practice. Such measures of leader attributes and skills as used in this study could be combined with more discrete measures of organizational performance to further illuminate the effect of leader style on outcomes. Further, thousands of public health leaders have been trained in state, territorial, regional, and national public health leadership institutes under the reasonable assumption that such training and development efforts improve the practice of public health. The Centers for Disease Control and Prevention (CDC), the Health Research Services Administration (HRSA), and others have invested tens of millions of dollars in these activities over the past two decades. As funding becomes increasingly limited and risks being diverted from leadership development activities, measures should be developed to objectively evaluate such activities. These measures should examine improvements in organizational financial performance, staff turnover, employee satisfaction, or other common and consistent metrics. Research into these topics should contribute substantively to the pillars of workforce research and development: enumeration, competency, and capacity.

### Limitations

Quantitative limitations include a small sample size and the use of self-reported data. Since responses were identifiable to the researcher and since an interpretive report was to be provided, it is plausible that participants may have been motivated to over-report what they felt were positive characteristics while under-reporting what they believed to be negative characteristics of leaders. Further, the sample of participants was randomly selected from a pre-identified pool of health department directors. It is possible that since most of the participants were white, female, and identified themselves as leading rural health departments, that these demographic factors have a significant influence on the styles of leadership practiced, preferred, or perceived to contribute to effective leadership outcomes. We did not examine how our sample compared to all US local health department directors. However, by focusing on non-urban directors and randomizing the selection, we believe our sample to be fairly representative. Sampling bias is a concern for this study, but one we accepted given the mixed-method nature of the overall study. The purpose of this study was not only to measure leadership styles, as described in this study, but also to purposefully examine transformational and transactional leaders in context. We note that caution should be taken when interpreting findings since while statistical significance was found for many measures, the small sample size and quasi-random sampling limit the generalizability of findings.

## Conclusion

In a time when healthcare, public health, and population health issues are being transformed, the critical importance of more fully understanding public health leadership cannot be understated. This study offers one quantitative perspective on the role of transformational leadership qualities in public health. Clearly, more detailed and in depth understanding is needed if we are to inform our educational and professional workforce development models related to public health leadership. Additional findings from a qualitative companion study are presented in the adjoining Part 2 paper.

## Conflict of Interest Statement

The authors declare that the research was conducted in the absence of any commercial or financial relationships that could be construed as a potential conflict of interest.
